# *E. coli* Infection Modulates the Pharmacokinetics of Oral Enrofloxacin by Targeting P-Glycoprotein in Small Intestine and CYP450 3A in Liver and Kidney of Broilers

**DOI:** 10.1371/journal.pone.0087781

**Published:** 2014-01-31

**Authors:** Mengjie Guo, Yong Sun, Yu Zhang, Shamsuddin Bughio, Xiaohua Dai, Weilong Ren, Liping Wang

**Affiliations:** 1 Laboratory of Veterinary Pharmacology and Toxicology, College of Veterinary Medicine, Nanjing Agricultural University, Nanjing, Jiangsu Province, PR China; 2 College of Food Science and Pharmacy, Xinjiang Agricultural University, Urumqi, China; Virginia Tech, United States of America

## Abstract

P-glycoprotein (P-gp) expression determines the absorption, distribution, metabolism and excretion of many drugs in the body. Also, up-regulation of P-gp acts as a defense mechanism against acute inflammation. This study examined expression levels of *abcb1* mRNA and localization of P-gp protein in the liver, kidney, duodenum, jejunum and ileum in healthy and *E. coli* infected broilers by real time RT-PCR and immunohistochemistry. Meanwhile, pharmacokinetics of orally administered enrofloxacin was also investigated in healthy and infected broilers by HPLC. The results indicated that *E. coli* infection up-regulated expression of *abcb1* mRNA levels significantly in the kidney, jejunum and ileum (*P*<0.05), but not significantly in the liver and duodenum (*P*>0.05). However, the expression level of *CYP 3A37* mRNA were observed significantly decreased only in liver and kidney of *E. coli* infected broilers (*P*<0.05) compared with healthy birds. Furthermore, the infection reduced absorption of orally administered enrofloxacin, significantly decreased C_max_ (0.34 vs 0.98 µg mL^−1^, *P* = 0.000) and AUC_0-12h_ (4.37 vs 8.88 µg mL^−1^ h, *P* = 0.042) of enrofloxacin, but increased T_max_ (8.32 vs 3.28 h, *P* = 0.040), T_1/2a_(2.66 vs 1.64 h^−1^, *P* = 0.050) and V/F (26.7 vs 5.2 L, *P* = 0.040). Treatment with verapamil, an inhibitor of P-gp, significantly improved the absorption of enrofloxacin in both healthy and infected broilers. The results suggest that the *E. coli* infection induces intestine P-gp expression, altering the absorption of orally administered enrofloxacin in broilers.

## Introduction

Colibacillosis is a common disease in poultry, affecting the growth of broilers and also the physiological function of the small intestine in the chickens. Fluoroquinolones have been successfully used to treat colibacillosis in poultry, among which enrofloxacin is most frequently applied [1]. Pharmacokinetic studies of enrofloxacin, which were performed in pets and food-producing animals under healthy conditions via various routes of administration, show that enrofloxacin exhibits good absorption, high bioavailability, large volume of distribution and low protein-binding [2], [3], [4]. Enrofloxacin is metabolized to ciprofloxacin via de-ethylation of the ethyl group on the piperazine ring [5] and mainly excreted as a parent drug and its metabolite by glomerular filtration and tubular secretion in the kidney, however, the rate of deethylation is various in different species[6], [7].

P-glycoprotein (P-gp) is encoded by *abcb1* gene, and belongs to ATP-binding cassette (ABC) superfamily, which is involved in drug transport [8]. P-gp, along with CYP 450 enzymes, also plays a key role in determining the dispositions of a variety of drugs in tissues [9]. *In vitro* and *in vivo* studies have demonstrated that P-gp can modulate the pharmacokinetics of human medicines, resulting in drug-drug interactions of structurally diverse compounds [10], [11]. Accumulating evidence implicates that many veterinary drugs, such as ivermectin, macrolides and fluoroquinoles, are also the substrates of P-gp [12], [13], [14]. Therefore, there are increasing attentions to the role of P-gp together with CYP450 in veterinary therapy [14]. It is well known that the expression of ABC transport proteins is regulated by a variety of factors, including pathological conditions, and in particular inflammatory reaction to infections [1]. However, it is not well understood whether modulation of their expression may subsequently affect the pharmacokinetics of drugs, altering the efficacy and toxicity of the drugs in animals [1], [14]. The pharmacokinetics of enrofloxacin has been extensively studied in broilers [15], but little is known about the relationship between the pharmacokinetics of enrofloxacin and the expression levels of P-gp and CYP450 enzymes in broilers, particularly following *E. coli* infection.

The present study examined the effects of *E. coli* infection on the expression levels of P-gp and CYP3A (cytochrome P450, family 3, subfamily A) in the liver, kidney and small intestine to clarify whether different expression levels of P-gp and CYP3A affect intestinal absorption, biliary secretion and kidney excretion of enrofloxacin in broilers. Furthermore, we investigated the influence of verapamil, an inhibitor of P-gp, on the absorption of enrofloxacin. The results indicate that *E. coli* infection modulates the pharmacokinetics of orally administered enrofloxacin by increasing intestinal P-gp expression and decreasing CYP3A expression in the liver and kidney of broilers.

## Materials and Methods

### Animals and reagents

Ross 308 broilers (one-day old, male and female randomly) were purchased from a local commercial poultry farm (Nanjing, China). All birds were kept at 25°C, had free access to standard commercial feed (without additives) and water, and treated following the protocol approved by Nanjing Agricultural University Animal Care and Use Committee. Before starting the experiment at the age of 4-week old, all broilers were verified free from colibacillosis. Mouse monoclonal anti-P-gp (C219) antibody, used for immunohistochemistry (IHC), was from Covance (Princeton, New Jersey, USA). Rabbit anti-mouse IgG-horseradish peroxidase (HRP) was purchased from Boster (Wuhan, Hubei, China). Verapamil was purchased from Sigma (St. Louis, MO, USA) and enrofloxacin was bought from China Institute of Veterinary Drug Control. All other reagents were purchased commercially with highest quality.

### *E. coli* infection model

*Escherichia coli* (*E. coli*) O_2_ strain, isolated from the broilers with colibacillosis, was kindly provided by the Laboratory of the Microbiology, Nanjing Agricultural University. The strain was stored at −80°C prior to use. The day before infection, *E. coli* was inoculated onto LB agar and incubated at 37°C for 24 h. Then eight colonies were suspended in 10 ml of broth and incubated at 37°C for 6 h when its OD_600_ value was about 0.6, as quantified by ultraviolet spectrophotometry. At the age of 4-week old, each broiler was given 0.5 ml of the overnight culture containing 1.5×10^8^ colony forming units (cfu) by pectoral muscle injection. Some broilers exhibited typical clinical signs of colibacillosis, including respiratory stress, and white loose droppings within 12 h after inoculation. Tissue samples collected from the dead broilers at necropsy were then cultured to confirm that the strain used to inoculate the birds caused the mortalities. In this study, three groups of broilers were infected with *E. coli*, including one group for immunohistochemistry of P-gp and mRNA quantitation (*abcb1*), and two groups for pharmacokinetic analysis of enrofloxacin.

### RNA isolation and real-time RT-PCR

Real time RT-PCR was used to detect the Abcb1 and CYP3A mRNA expression levels in the liver, kidney and different parts of small intestine in healthy (n = 5) and *E. coli* infected (n = 5) broilers. Total RNA was isolated from individual tissues of all birds using Trizol Reagent (Takara, Tokyo, Japan) according to the manufacturer's instructions. All RNA samples were treated with 100 U DNase I (RNase Free, Takara, Tokyo, Japan) for 30 min at 37°C to ensure that the samples were free of genomic DNA contamination. The total RNA concentration was then quantified using a Nanodrop photometer (ND-1000 Spectrophotometer, Rockland, DE, USA). Ratios of the optical density (OD) values at 260/280 nm of all preparations were between 1.8 and 2.0. Each RNA sample was subjected to electrophoresis on a 1.4% agarose formaldehyde gel to verify its integrity. Single-stranded cDNAs were synthesized and real time PCR was performed, as described previously [16].Negative controls involved omission of RNA from the reverse transcription reactions and amplifications with specific primer/probe sets to confirm the lack of genomic DNA contamination. Primers specific for *abcb1*, CYP3A and β-actin were designed as described [1], [17] and commercially synthesized for real-time PCR analysis. Broiler β-actin was chosen as a housekeeping gene for normalization, based on experiments showing stable expression of β-actin mRNA in the small intestine, liver and kidney of broilers. The PCR products were sequenced to validate the identity of the amplicons. The 2^−ΔΔCt^ method [18] was used to analyze the real-time RT-PCR data.

### Immunohistochemistry

To study the localization and semi-quantitative protein expression of P-gp in the liver and small intestine of healthy and *E. coli* infected broilers at 4-week old, immunohistochemical staining was performed as described previously [19]. Briefly, the tissues from 5 healthy birds and 5 *E. coli* infected broilers were collected. The tissue-sections were prepared and incubated overnight at 4°C with the primary antibody (C219, monoclonal anti-P-gp, 1:20) and at 37°C for 1 h with the secondary antibody (rabbit anti-mouse IgG-HRP), and the P-gp immunoreactivity was visualized with DAB staining. Sections without incubation with the primary antibody served as negative controls. Three semi-quantitative measurements for P-glycoprotein staining were performed by two experimental pathologists in a double-blind analysis under a light microscope (BX45-DP72; OLYMPUS, Tokyo, Japan) equipped with Plan Apo objectives connected to a CCD camera (U-TV0.63XC; OLYMPUS, Tokyo, Japan) as previously described [19].

### Experimental design for pharmacokinetic analysis of enrofloxacin in broilers

Forty 28-day old broilers were randomly divided into 4 groups with 10 broilers in each group in this study. Group I birds received enrofloxacin orally with single dose of 10 mg/kg b.w. through crop tube gavages. Group II was pre-treated with verapamil (15 mg/kg b.w.) orally for 30 min, followed by oral administration of enrofloxacin with single dose of 10 mg/kg b.w as previously reported [19]. Group III was given with a single dose of enrofloxacin (10 mg/kg b. w) through oral administration after 12 h of *E. coli* challenge. Group IV, 30 min prior to enrofloxacin (10 mg/kg b. w.) oral administration, was treated with verapamil (15 mg/kg b. w.) orally following 12 h of *E. coli* challenge. Plasma samples for HPLC analysis were drawn in heparin tubes 20 min before and at 0.083, 0.25, 0.33, 0.5, 0.75, 1, 2, 3, 4, 6, 8 and 12 h following enrofloxacin administration in each group. The samples were placed on ice before transporting to the laboratory and centrifuged at 1 500 g for 10 min. The centrifuged plasma was harvested and aliquot for storage at −80°C before HPLC analysis.

### HPLC assay for detection of enrofloxacin in plasma of broilers

The plasma concentrations of enrofloxacin were detected through Agilent 1200 high-performance liquid chromatography (HPLC) system as described previously with minor modification [13], [20]. Briefly, the blood samples thawed out at room temperature and centrifuged at 2 000 g for 5 min, the supernatant (0.5 ml) was applied to acetonitrile and the organic and water phases were separated by centrifugation. The organic phase was evaporated to dryness under a nitrogen stream and the residue was re-suspended with mobile phase solution. Twenty microliters of the mixture was injected into the HPLC column. The composition of the mobile phase was 0.1 M phosphoric acid (adjust pH to 3.0 with triethylamine)/acetonitrile (84:16, v/v). Enrofloxacin plasma concentration was determined using a Waters e2695 HPLC system (Waters, Japan). HPLC analysis was performed on Kromasil C_18_ HPLC Columns (5 µm, 25 cm×4.6 mm). The flow rate of the mobile phase was set to 0.85 ml/min. UV absorbance was measured at 278 nm.

### Pharmacokinetic analysis

Pharmacokinetic calculations were performed on each individual set of data using 3p97 practical pharmacokinetic software (Version97, Chinese Pharmacologic Association, Beijing, China). The best fit of compartment model was determined according to the Akaike’s Information Criterion. The area under the concentration–time curve (AUC_0-12h_) was calculated according to the linear trapezoidal method.

### Data analysis

All data were presented as mean ± S.E.M., and analyzed by one-way ANOVA using SPSS 16.0 for Windows followed by a least-significant difference (LSD) test for individual comparisons. Values of mRNA abundance were expressed as the fold change relative to the average value of one group. Pharmacokinetic parameters of enrofloxacin were analyzed using student *t-*test for independent samples. The significance level was set at *P*<0.05.

## Results

### Clinical observations and pathology in *E. coli* infected broilers

For colibacillosis model, each broiler was inoculated with 0.5 ml of *E. coli* culture containing 1.5×10^8 ^cfu/ml by pectoral muscle injection. We found that within 12 h of inoculation, two broilers died of the infection and others showed clinical signs at different degrees. Some broilers exhibited typical clinical signs of colibacillosis, including respiratory stress, and white loose droppings. Necropsy lesions showed a layer of white cellulose pseudo-membranous covering the surface of the liver and heart. Also, severe bleeding and swelling were observed in most segments of the small intestine except duodenum. *E. coli* was also isolated in liver from the diseased birds.

### mRNA expression levels of *abcb1* and *CYP 3A37* in liver, kidney and small intestines in healthy and *E. coli* infected broilers

**Figure 1:**
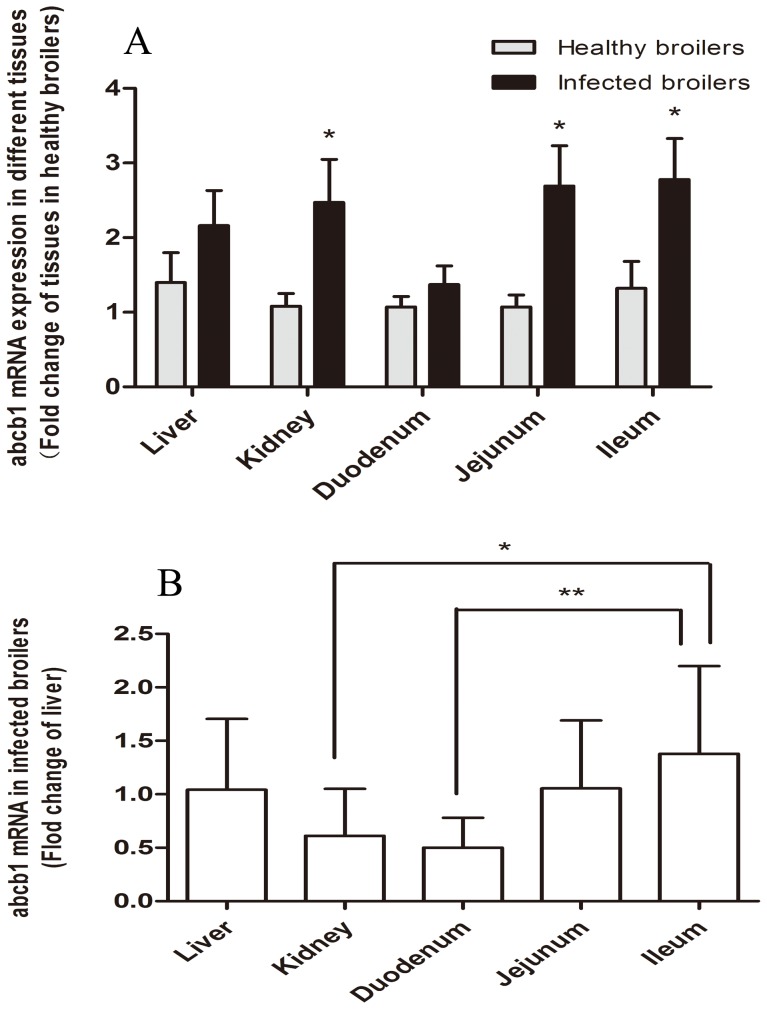
Expression levels of *abcb1* mRNA in broilers by real time RT PCR (n = 5). A: Differences of *abcb1* mRNA level in liver, jejunum, ileum and duodenum between healthy and *E. coli* infected broilers; B: Comparison of *abcb1* mRNA level in different tissues of infected broilers. β-actin was used as a reference gene for normalization. All data were presented as mean ± S.E.M. and analyzed by one-way ANOVA using SPSS 16.0 for Windows followed by a least-significant difference (LSD) test for individual comparisons. * *P*<0.05, ** *P*<0.01.

**Figure 2:**
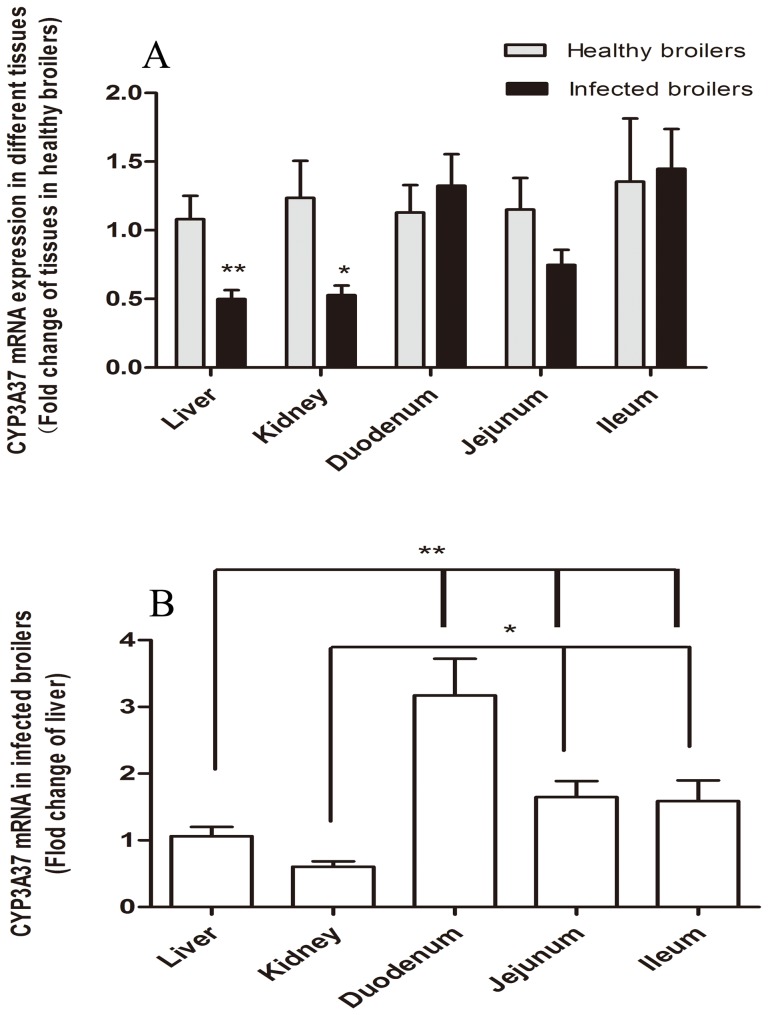
Expression levels of *CYP3A37* mRNA in broilers by real time RT PCR (n = 5). A: Differences of *CYP3A37* mRNA level in liver, jejunum, ileum and duodenum between healthy and *E. coli* infected broilers; B: Comparison of *CYP3A37* mRNA level in different tissues of infected broilers. β-actin was used as a reference gene for normalization. The results were expressed as the mean ± S.E.M. and analyzed by one-way ANOVA using SPSS 16.0 for Windows followed by a least-significant difference (LSD) test for individual comparisons. * *P*<0.05, ** *P*<0.01.

The expression level of P-gp encoding gene *abcb1* and *CYP 3A37* were detected by real time PCR with β-actin chosen as a housekeeping gene for normalization. Compared with the healthy group, the *abcb1* mRNA level in the kidney and small intestines was up-regulated after *E. coli* infection. As shown in Fig.1A, *abcb1* mRNA was detected at significantly higher levels in kidney (*P* = 0.032), jejunum (*P* = 0.017) and ileum (*P* = 0.046) of infected birds than that of healthy broilers, whereas the mRNA levels in duodenum (*P* = 0.349) and liver (*P* = 0.264) were not significantly changed in *E. coli* infected broilers, though an increasing trend was observed. In different tissues of infected broilers, the highest level of *abcb1* mRNA was observed in ileum, and followed by jejunum, liver, kidney and duodenum (Fig.1B). However, the expression level of *CYP 3A37* were observed significantly decreased only in liver and kidney of *E. coli* infected broilers compared with healthy birds, which was in contrast with the pattern of *abcb1* expression level (Fig. 2A). In each tested tissues, infection sharply decreased CYP *3A37* and the level of CYP *3A37* in liver even lower than that of small intestine (Fig. 2B).

### Effect of *E. coli* infection on P-gp localization and expression

**Figure 3:**
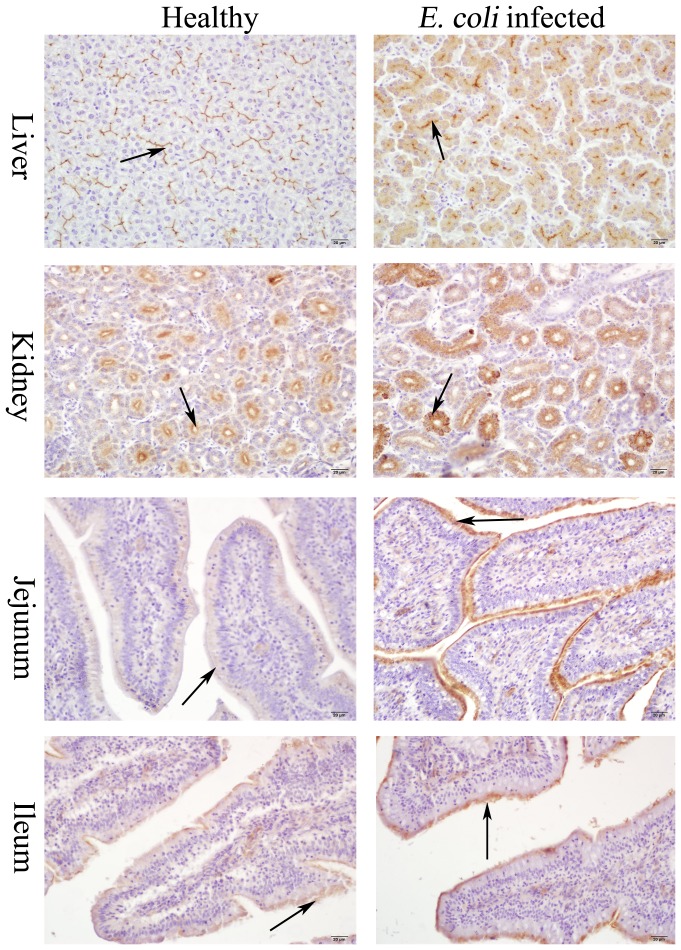
Immunohistochemical staining of P-glycoprotein in different tissues of healthy and *E. coli* infected broilers. Mouse monoclonal anti-P-gp antibody (C219) was used to detect P-gp. These figures are representatives of typical samples from 5 broilers in each group. The arrow showed the positive staining of P-gp. Magnifications are indicated by bar.

**Figure 4:**
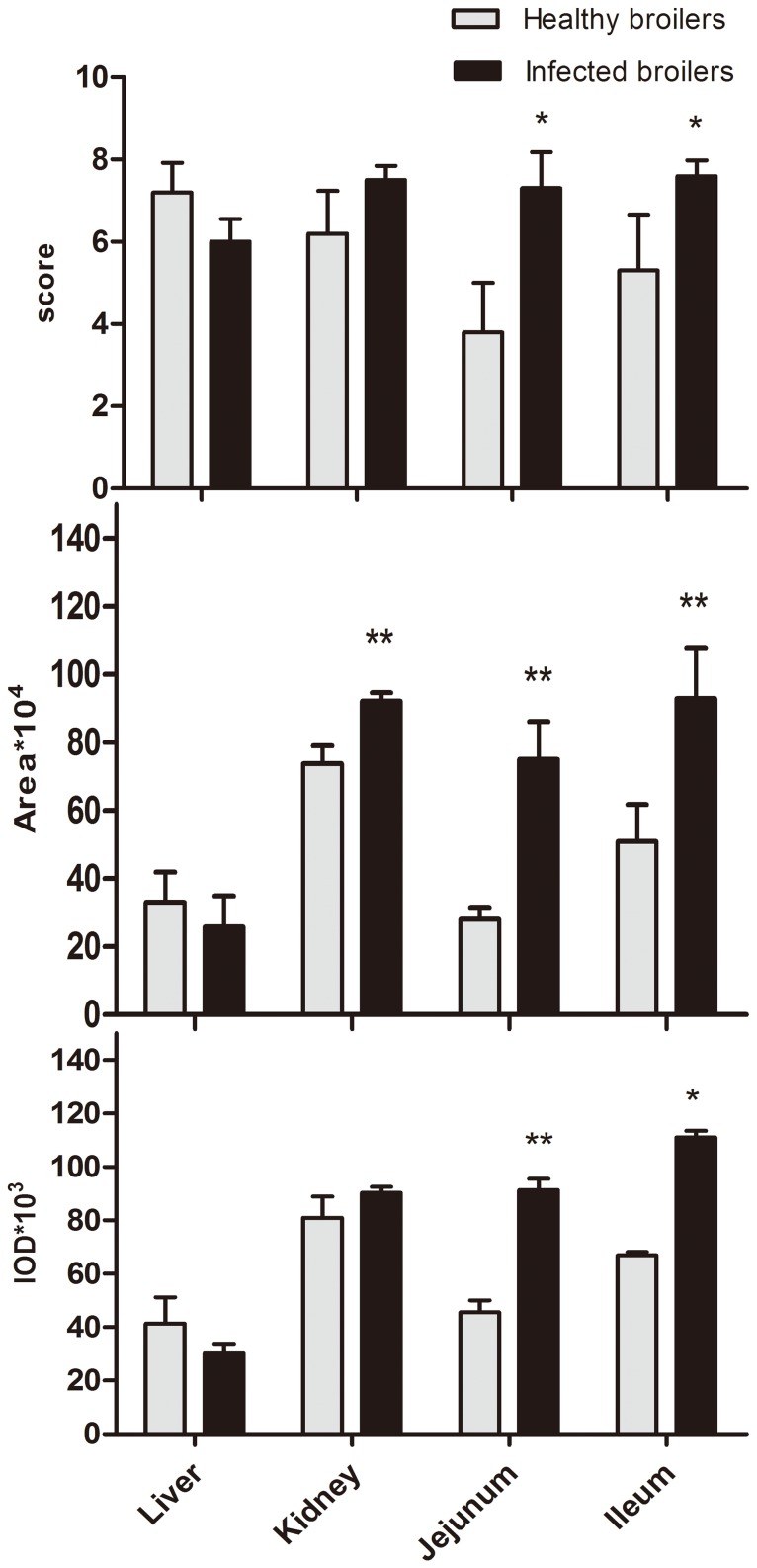
Semi-quantification of P-gp in both healthy and *E. coli* infected broilers. The intensity of specific P-gp staining was evaluated by measuring the IOD, area of positive staining and scores of its expression respectively. Digital images of five sections from each broiler of the surface epithelium area were evaluated. Each section was divided into five sub-areas, and the IOD and positive area of the staining was analyzed using the Image-Pro Plus 4.1. software and scores were analyzed from 0 to 4 to estimate the positive staining in different areas under study.*P<0.05, **P<0.01.

To date, there are no reports on the effects of *E. coli* infection on the localization and expression level of P-gp in the small intestine, liver and kidney. In this study, IHC method was used to determine the localization and expression level of P-gp in broilers. The results showed that no background staining was observed in the negative controls (data not shown). Positive staining was detected in liver, kidney, jejunum and ileum of healthy and *E. coli* infected birds as shown in Fig.3. In healthy birds, immunoreactivity of P-gp was both visualized on the apical surface of the enterocytes of ileum and jejunum, while in infected broilers, positive staining of P-gp remained localized in the membrane of the enterocytes but the intensity was significantly increased. Remarkable P-gp immunostaining was observed in the bile canalicular membranes of the hepatocytes in healthy broilers. However, P-gp was internalized into the cytoplasm away from the biliary membrane in liver from *E. coli* infected broilers. Similarly in kidney, the main staining was observed in the apical plasma membranes of proximal tubule cells of healthy broilers, but a punctuate labeling for P-gp was distributed widely throughout the cytoplasm in the kidneys of infected broilers. To validate our immunohistochemical results, we semi-quantified the stained liver, kidneys and small intestines from healthy and *E. coli* infected broilers using Image-Pro Plus 4.1 software. For each experimental group, IOD, positive area and score for 25 samples from each bird was analyzed, and a comparison was made between the average values obtained (Fig. 4). Compared with healthy birds in control group, *E. coli* infection treatment significantly enhanced (*P*<0.05) P-gp staining of jejunum and ileum via IOD, positive area and scores evaluation. Though the total P-gp level was increased in liver and kidney, the level in the bile canalicular membranes and apical plasma membranes of proximal tubule cells was not significantly changed via IOD, positive area and score estimates. In kidney, the area of positive staining was enhanced, but IOD and score were not changed. Quantification of P-gp staining in intestine provided additional validity to our studies at protein level, which was coincident with the trend of changes in mRNA expression changes between healthy and infected birds.

### Pharmacokinetics of orally administered enrofloxacin in infected and healthy broilers

**Figure 5:**
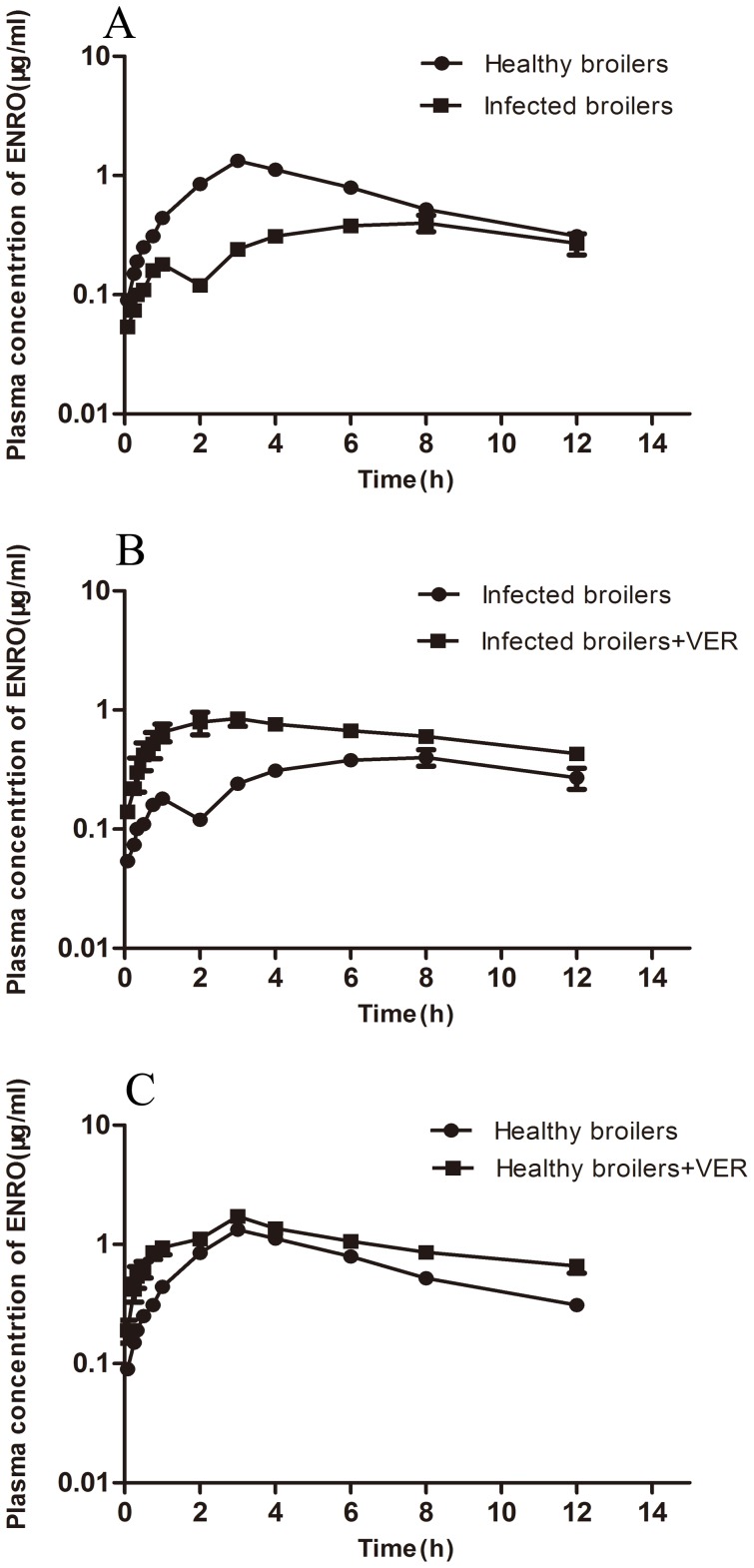
Mean plasma enrofloxacin concentrations in broilers after enrofloxacin administration. A: enrofloxacin alone by single oral administration (10 mg/kg) to healthy (n = 10) and infected (n = 10) broilers; B: enrofloxacin after a single oral administration of 10 mg/kg alone (n = 10) and co-administration of verapamil (15 mg/kg, n = 10) to *E. coli* infected broilers C: enrofloxacin after a single oral administration of 10 mg/kg alone (n = 10) and co-administration of verapamil (15 mg/kg, n = 10) to healthy broilers. Each point represents the mean ±S.E.M. of 10 broilers.

To further confirm whether P-gp and CYP3A expression levels are the main factors affecting the pharmacokinectics of enrofloxacin in broilers after infection, both infected and healthy broilers were administered a single dose of enrofloxacin orally. Following oral administration, a one-compartment open model was found to best fit concentration-time data of enrofloxacin. The plasma concentration *vs* time curves for enrofloxacin are shown in Fig.5A. Pharmacokinetic parameters of enrofloxacin in broilers before and after inoculation of *E. coli* are presented in Table 1. Lower plasma concentrations of enrofloxacin were found in infected broilers. Compared to healthy broilers, infected ones were observed with significantly decreased *C_max_* (*P* = 0.000) and *AUC_0-12h_* (*P* = 0.042, absorptive phase) as well as obviously longer *T_max_* (*P* = 0.040)and *T_1/2a_* (*P* = 0.045), indicating that the absorption of enrofloxacin was slower and inhibited. Consistent with these findings, higher mRNA expression of P-gp was observed in the small intestine in broilers after *E. coli* infection. However, infected birds demonstrated lowered *Cl/F* (*P* = 0.038) but higher *T_1/2e_* (*P* = 0.000) and volume of distribution (*P* = 0.040), compared with healthy birds, suggesting that the clearance of enrofloxacin was also inhibited by *E. coli* infection.

**Table 1:** Parameters of oral enrofloxacin in healthy and infected broilers (mean ± S.E.M., n = 10).

Parameters	Healthy broilers	Infected broilers	*P* value
Ke (h^−1^)	0.21±0.007	0.024±0.003^**^	0.000
Ka (h^−1^)	0.43±0.03	0.46±0.09	0.851
T*_1/2a_* (h)	1.64±0.1	2.66±0.88*	0.045
T*_1/2e_* (h)	3.36±0.12	24.6±1.69^**^	0.000
T_max_ (h)	3.28±0.11	7.1±0.91*	0.040
C_max_ (µg mL^−1^)	0.98±0.03	0.34±0.04^**^	0.000
AUC_0-12h_ (µg mL^−1^ h)	8.88±0.57	4.37±0.82*	0.042
V/F (L)	5.2±0.18	26.7±4.03*	0.040
*Cl*/F (mL/min)	1.08±0.04	0.6±0.1*	0.038

* *P*<0.05, ** *P*<0.01 significant difference vs. healthy broliers. *K_e:_*,elimination rate constant; *K_a_*, absorption rate constant; *t*_1/2a_, the absorption half-life; *t*_1/2e_, the elimination half-life; T_max_, the time to reach peak concentration; C_max_, the peak concentration; AUC_0-12h_, the area under the plasma concentration-time curve from zero to 12h; V/F, volume of distribution/F, where F is the faction of dose absorbed; Cl/F, Clearance/F, where F is the faction of dose absorbed.

### Effect of verapamil on pharmacokinetics of orally administered enrofloxacin

To investigate whether *E. coli* infection modulates the pharmacokinetics of oral enrofloxacin by targeting the intestinal P-glycoprotein expression, the pharmacokinetics of enrofloxacin was also studied after exposure to verapamil (a potent and selective inhibitor of P-gp inhibitor) for 30 min in both healthy and infected groups. The plasma concentration of enrofloxacin in the presence or absence of oral verapamil (15 mg/kg b.w.) was measured after oral administration of enrofloxacin (10 mg/kg). The plasma concentration-time profiles are shown in Fig.5B and 5C. According to the pharmacokinetic parameters presented in Table 2 and 3, verapamil showed a non-negligible effect on the pharmacokinetic of enrofloxacin. After treated with verapamil, C_max_ (1.46- and 2.94-fold) and AUC _0-12h_ (1.33-fold and 2.39-fold) of enrofloxacin increased in healthy and infected broilers, respectively. Meanwhile, the T_max_ of enrofloxacin was significantly short and Ka of enrofloxacin was significantly higher when P-gp was inhibited with verapamil. The plasma concentration of enrofloxacin was altered in both healthy and *E. coli* infected broilers, which was prevented by verapamil, indicating that the absorption was P-gp-dependent.

**Table 2:** Parameters of oral enrofloxacin in healthy broilers with and without verapamil (mean±S.E.M., n = 10).

Parameters	ENRO	ENRO+VER	*P* value
Ke (h^−1^)	0.21±0.007	0.09±0.01^**^	0.003
Ka (h^−1^)	0.43±0.03	1.02±0.17	0.072
T_1/2a_ (h)	1.64±0.1	0.79±0.1*	0.010
T_1/2e_ (h)	3.36±0.12	8.43±1.04*	0.023
T_max_ (h)	3.28±0.11	2.86±0.29	0.429
C_max_ (µg mL^−1^)	0.98±0.03	1.43±0.11	0.051
AUC_0-12h_ (µg mL^−1^ h)	8.88±0.57	11.79±1.24*	0.042
*V/F*( L )	5.2±0.18	5.98±0.6	0.466
*Cl*/F(mL/min)	1.08±0.04	0.51±0.03^**^	0.000

* *P*<0.05, **P<0.01 significant difference between parameters of enrofloxacin in the presence and absence of verapamil in healthy broilers.

**Table 3:** Parameters of oral enrofloxacin in infected broilers with and without verapamil (mean±S.E.M., n = 10).

Parameters	ENRO	ENRO+VER	*P* value
Ke (h^−1^)	0.024±0.003	0.1±0.009^**^	0.002
Ka (h^−1^)	0.46±0.09	0.88±0.13	0.142
T_1/2a_ (h)	2.66±0.88	0.95±0.16	0.273
T_1/2e_ (h)	24.6±1.69	6.92±0.73^**^	0.002
T_max_	7.1±0.91	3.13±0.45*	0.049
C_max_ (µg mL^−1^)	0.34±0.04	1.0±0.11*	0.017
AUC_0-12h_ (µg mL^−1^ h)	4.37±0.82	10.43±0.3*	0.019
*V/F*( L )	26.7±4.03	12.12±2.83	0.114
*Cl*/F (mL/min)	0.6±0.1	1.21±0.27	0.232

* *P*<0.05, **P<0.01 significant difference between parameters of enrofloxacin in the presence and absence of verapamil in infected broilers.

## Discussion

In this study, we focused on clarifying whether *E. coli* infection affects the pharmacokinetic parameters of enrofloxacin by modifying P-gp expression levels in the tissues of 4-week old broilers. The main finding of the present study is that *E. coli* infection resulted in an increased expression of *abcb1* and P-gp in the small intestine and kidney. The observed changes in the expression level of P-gp mRNA/protein suggest that *E. coli* infection affected P-gp expression at the transcriptional and translation levels. Likely, *E. coli* infection-induced change in the absorption of enrofloxacin was due to increased expression of P-gp in the small intestine. This is also supported by the data that decreased C_max_ and AUC_0-12h_ of enrofloxacin in *E. coli* infected broilers was paralleled to increased *abcb1* mRNA and P-gp protein in jejunum and ileum. The decrease in plasma level of enrofloxacin was especially evident shortly after enrofloxacin administration, suggesting that the intestinal uptake was inhibited. Though the absorption of enrofloxacin declined, volume of distribution increased and terminal half-life became longer, which might be associated with decreased CYP_450_ level or attenuated function of P-gp in liver and kidney, leading to decreased clearance of enrofloxacin in infected birds compared with healthy ones.

Injection of bacterial lipopolysaccharide (LPS) is a widely used model of inflammation and is well characterized regarding its effect on P-gp expression. LPS-medicated induction of pro-inflammatory cytokines activates a variety of cell signaling molecules, including c-Jun N-terminal kinase (JNK) and nuclear factor kappa B (NF-κB) [21]. Activation of JNK leads to suppression of pregnane X receptor (PXR) and constitutive androstane receptor (CAR), and subsequently their target genes including *abcb1* and *CYP450* are rapidly and significantly repressed [22]. Our results showed that *E. coli* infection up-regulated P-gp expression in broilers, which is much different from that in mammals where LPS induced down-regulation of P-gp during the acute phase response via nuclear receptors [23], [24], [25]. Possibly, this is due to the different types of inflammation inducers used (LPS vs. *E. coli* bacteria); *E. coli* may induce more cytokines mediating more cell signaling pathways. It has been pointed out that LPS is not a generic substitute for bacterial infection to study its effect on P-gp expression, function and on drug pharmacokinetics [26]. Alternatively, this might be due to different species used (chickens vs. rats). Till now, there are few studies available about *E. coli* infection or LPS on the P-gp expression in poultry. Clearly, further research is required to address this issue. Nevertheless, our results are in line with other findings [24], [25], [26]. It has been described that tumor necrosis factor-α (TNF-α) induces *bAbcb1*mRNA and protein in mammary epithelial monolayer and BME-UV cells as well as in mice, and Shiga-like toxin II increases P-gp expression in mouse brain [27], [28], [29]. This increase involves signaling through the TNF-R1 receptor, ETA and ETB receptors, NOS, PKC and the transcription factor NF-κB [24], [25], [26]. Taken together, our and other findings suggest novel signaling pathways through which inflammation can up-regulate P-gp expression and activity.

The increase in the levels of *abcb1* by *E. coli* infection following therapy was likely to reflect a beneficial effect on the bird, as this up-regulation might improve the barrier function of the gastro-intestinal tract through effluxing toxins, including LPS, and enhance the resistance against further infections by viruses and bacteria [30], [31], [32], [33], [34], [35]. However, meanwhile, induction of intestinal P-gp by *E. coli* infection also reduced absorption of orally administered enrofloxacin. Therefore, when antimicrobial agents are used in clinics to treat *E. coli* infections, both of the above two aspects should be taken into consideration in order to reach effective concentrations of the drugs in animal body.

We found that the efflux rate of enrofloxacin through bile canalicular and tubular secretion was not paralleled to up-regulation trend of *abcb1* mRNA in liver and kidney in infected broilers based on the longer half-life time and volume of distribution (7.32- and 5.13-fold more than that of healthy birds, respectively). The present experiments were designed to test the hypothesis that loss of P-gp transport activity of bile canaliculus and tubule is accompanied by a shift in transporter protein from the luminal membrane to a compartment where it can no longer function as an efflux transporter. Therefore, we further detected the expression of P-gp and its localization using IHC method. The images showed that P-gp was internalized into the cytoplasm away from the biliary membrane in the liver and apical plasma membranes of proximal tubule in the kidney of *E. coli* infected broilers but not in healthy birds (showed by Fig. 3). This indicated that *E. coli* infection might have adversely affected protein trafficking as P-gp went to the cell surface or led to mislocalization of P-gp on the plasma membrane, resulting in a reduced capacity to transport substrate. The result was similar to the finding that LPS can induce the translocation of MRP2 from the canalicular membrane into cytoplasmic membrane vesicles by failure to anchor properly the membrane protein to the cytoskeleton, resulting in mislocalization in the cell membrane [36], [37]. Recent reports also reveal that the mechanism of rapid loss of P-gp activity in response to TNF-α/PKCbI or VEGF/Src *in vitro* and *in vivo* is posttranslational and dependent on protein phosphorylation-based signaling [38], [39]. However, it remains unclear why the P-gp localization in the enterocytes of small intestine is not changed in response to *E. coli* infection.

In summary, here, for the first time, we showed that *E. coli* infection increased mRNA and P-gp expression in the intestine, but decreased CYP3A expression in the liver and kidney in broilers. Consequently, this modulated the pharmacokinetics of enrofloxacin in broilers. The findings provide strong evidence that it is essential to optimize the usage and dosage of enrofloxacin in *E. coli* infected broilers for a better treatment of colibacillosis. Also, from the present study, we should be aware that a healthy animal model cannot substitute for an infection animal model to evaluate the pharmacokinetics of enrofloxacin in broilers.

## References

[1] Haritova AM, Rusenova NV, Rusenov AG, Schrickx J, Lashev LD, et al (2008) Effects of fluoroquinolone treatment on MDR1 and MRP2 mRNA expression in Escherichia coli-infected chickens. Avian Pathol 37: 465–470. 18663652 10.1080/03079450802272945

[2] Bregante MA, Saez P, Aramayona JJ, Fraile L, Garcia MA, et al (1999) Comparative pharmacokinetics of enrofloxacin in mice, rats, rabbits, sheep, and cows. Am J Vet Res 60: 1111–1116. 10490081

[3] Elmas M, Tras B, Kaya S, Bas AL, Yazar E, et al (2001) Pharmacokinetics of enrofloxacin after intravenous and intramuscular administration in Angora goats. Can J Vet Res 65: 64–67. 11227198 PMC1189645

[4] Cox SK, Cottrell MB, Smith L, Papich MG, Frazier DL, et al (2004) Allometric analysis of ciprofloxacin and enrofloxacin pharmacokinetics across species. J Vet Pharmacol Ther 27: 139–146. 15189299 10.1111/j.1365-2885.2004.00560.x

[5] Tyczkowska K, Hedeen KM, Aucoin DP, Aronson AL (1989) High-performance liquid chromatographic method for the simultaneous determination of enrofloxacin and its primary metabolite ciprofloxacin in canine serum and prostatic tissue. J Chromatogr 493: 337–346. 2584299 10.1016/s0378-4347(00)82739-5

[6] Vancutsem PM, Babish JG, Schwark WS (1990) The fluoroquinolone antimicrobials: structure, antimicrobial activity, pharmacokinetics, clinical use in domestic animals and toxicity. Cornell Vet 80: 173–186. 2180631

[7] McKellar QA, Benchaoui HA (1996) Avermectins and milbemycins. J Vet Pharmacol Ther 19: 331–351. 8905567 10.1111/j.1365-2885.1996.tb00062.x

[8] Sharom FJ (2008) ABC multidrug transporters: structure, function and role in chemoresistance. Pharmacogenomics 9: 105–127. 18154452 10.2217/14622416.9.1.105

[9] Russel FGM (2010) Transporters: Importance in Drug Absorption, Distribution, and Removal. 27–49.

[10] Lin JH, Yamazaki M (2003) Role of P-glycoprotein in pharmacokinetics: clinical implications. Clin Pharmacokinet 42: 59–98. 12489979 10.2165/00003088-200342010-00003

[11] Cox DS, Scott KR, Gao H, Eddington ND (2002) Effect of P-glycoprotein on the pharmacokinetics and tissue distribution of enaminone anticonvulsants: analysis by population and physiological approaches. J Pharmacol Exp Ther 302: 1096–1104. 12183668 10.1124/jpet.102.035436

[12] Molento MB, Lifschitz A, Sallovitz J, Lanusse C, Prichard R (2004) Influence of verapamil on the pharmacokinetics of the antiparasitic drugs ivermectin and moxidectin in sheep. Parasitol Res 92: 121–127. 14634800 10.1007/s00436-003-1022-3

[13] Pulido MM, Molina AJ, Merino G, Mendoza G, Prieto JG, et al (2006) Interaction of enrofloxacin with breast cancer resistance protein (BCRP/ABCG2): influence of flavonoids and role in milk secretion in sheep. J Vet Pharmacol Ther 29: 279–287. 16846465 10.1111/j.1365-2885.2006.00744.x

[14] Schrickx JA, Fink-Gremmels J (2008) Implications of ABC transporters on the disposition of typical veterinary medicinal products. Eur J Pharmacol 585: 510–519. 18417119 10.1016/j.ejphar.2008.03.014

[15] Bugyei K, Black WD, McEwen S (1999) Pharmacokinetics of enrofloxacin given by the oral, intravenous and intramuscular routes in broiler chickens. Can J Vet Res 63: 193–200. 10480461 PMC1189547

[16] Li R, Hu Y, Ni Y, Xia D, Grossmann R, et al (2011) Leptin stimulates hepatic activation of thyroid hormones and promotes early posthatch growth in the chicken. Comp Biochem Physiol A Mol Integr Physiol 160: 200–206. 21679771 10.1016/j.cbpa.2011.06.001

[17] Ourlin JC, Baader M, Fraser D, Halpert JR, Meyer UA (2000) Cloning and functional expression of a first inducible avian cytochrome P450 of the CYP3A subfamily (CYP3A37). Arch Biochem Biophys 373: 375–384. 10620362 10.1006/abbi.1999.1566

[18] Livak KJ, Schmittgen TD (2001) Analysis of relative gene expression data using real-time quantitative PCR and the 2(-Delta Delta C(T)) Method. Methods 25: 402–408. 11846609 10.1006/meth.2001.1262

[19] Guo M, Bughio S, Sun Y, Zhang Y, Dong L, et al (2013) Age-Related P-Glycoprotein Expression in the Intestine and Affecting the Pharmacokinetics of Orally Administered Enrofloxacin in Broilers. PLoS One 8: e74150. 24066110 10.1371/journal.pone.0074150 PMC3774662

[20] Idowu OR, Peggins JO (2004) Simple, rapid determination of enrofloxacin and ciprofloxacin in bovine milk and plasma by high-performance liquid chromatography with fluorescence detection. J Pharm Biomed Anal 35: 143–153. 15030889 10.1016/j.jpba.2004.01.006

[21] Ghose R, Guo T, Vallejo JG, Gandhi A (2011) Differential role of Toll-interleukin 1 receptor domain-containing adaptor protein in Toll-like receptor 2-mediated regulation of gene expression of hepatic cytokines and drug-metabolizing enzymes. Drug Metab Dispos 39: 874–881. 21303924 10.1124/dmd.110.037382 PMC3082375

[22] Ghose R, Guo T, Haque N (2009) Regulation of gene expression of hepatic drug metabolizing enzymes and transporters by the Toll-like receptor 2 ligand, lipoteichoic acid. Arch Biochem Biophys 481: 123–130. 18940178 10.1016/j.abb.2008.10.003 PMC2635100

[23] Cherrington NJ, Slitt AL, Li N, Klaassen CD (2004) Lipopolysaccharide-mediated regulation of hepatic transporter mRNA levels in rats. Drug Metab Dispos 32: 734–741. 15205389 10.1124/dmd.32.7.734

[24] Moriguchi J, Kato R, Nakagawa M, Hirotani Y, Ijiri Y, et al (2007) Effects of lipopolysaccharide on intestinal P-glycoprotein expression and activity. Eur J Pharmacol 565: 220–224. 17399699 10.1016/j.ejphar.2007.02.058

[25] Tomita M, Kanbayashi A, Murata H, Tanaka A, Nakaike M, et al (2010) Effect of lipopolysaccharide on P-glycoprotein-mediated intestinal and biliary excretion of rhodamine123 in rats. Int J Pharm 392: 35–41. 20363306 10.1016/j.ijpharm.2010.03.019

[26] Post LO, Farrell DE, Cope CV, Baker JD, Myers MJ (2003) The effect of endotoxin and dexamethasone on enrofloxacin pharmacokinetic parameters in swine. J Pharmacol Exp Ther 304: 889–895. 12538847 10.1124/jpet.102.042416

[27] Zhao YL, Du J, Kanazawa H, Cen XB, Takagi K, et al (2002) Shiga-like toxin II modifies brain distribution of a P-glycoprotein substrate, doxorubicin, and P-glycoprotein expression in mice. Brain Res 956: 246–253. 12445692 10.1016/s0006-8993(02)03546-1

[28] Quesnell RR, Erickson J, Schultz BD (2007) Apical electrolyte concentration modulates barrier function and tight junction protein localization in bovine mammary epithelium. Am J Physiol Cell Physiol 292: C305–318. 16885391 10.1152/ajpcell.00567.2005

[29] Al-Bataineh MM, Van Der Merwe D, Schultz BD, Gehring R (2012) Molecular and functional identification of organic anion transporter isoforms in cultured bovine mammary epithelial cells (BME-UV). J Vet Pharmacol Ther 35: 209–215. 21623837 10.1111/j.1365-2885.2011.01309.x PMC3165114

[30] Panwala CM, Jones JC, Viney JL (1998) A novel model of inflammatory bowel disease: mice deficient for the multiple drug resistance gene, mdr1a, spontaneously develop colitis. J Immunol 161: 5733–5744. 9820555

[31] Lee CG, Ramachandra M, Jeang KT, Martin MA, Pastan I, et al (2000) Effect of ABC transporters on HIV-1 infection: inhibition of virus production by the MDR1 transporter. FASEB J 14: 516–522. 10698967 10.1096/fasebj.14.3.516

[32] Raviv Y, Puri A, Blumenthal R (2000) P-glycoprotein-overexpressing multidrug-resistant cells are resistant to infection by enveloped viruses that enter via the plasma membrane. FASEB J 14: 511–515. 10698966 10.1096/fasebj.14.3.511

[33] Schwab D, Fischer H, Tabatabaei A, Poli S, Huwyler J (2003) Comparison of in vitro P-glycoprotein screening assays: recommendations for their use in drug discovery. J Med Chem 46: 1716–1725. 12699389 10.1021/jm021012t

[34] Leslie EM, Deeley RG, Cole SP (2005) Multidrug resistance proteins: role of P-glycoprotein, MRP1, MRP2, and BCRP (ABCG2) in tissue defense. Toxicol Appl Pharmacol 204: 216–237. 15845415 10.1016/j.taap.2004.10.012

[35] Crowe A, Bebawy M (2012) ABCB1 (P-glycoprotein) reduces bacterial attachment to human gastrointestinal LS174T epithelial cells. Eur J Pharmacol 689: 204–210. 22683872 10.1016/j.ejphar.2012.05.047

[36] Elferink MG, Olinga P, Draaisma AL, Merema MT, Faber KN, et al (2004) LPS-induced downregulation of MRP2 and BSEP in human liver is due to a posttranscriptional process. Am J Physiol Gastrointest Liver Physiol 287: G1008–1016. 15205115 10.1152/ajpgi.00071.2004

[37] Apodaca G (2001) Endocytic traffic in polarized epithelial cells: role of the actin and microtubule cytoskeleton. Traffic 2: 149–159. 11260520 10.1034/j.1600-0854.2001.020301.x

[38] Hawkins BT, Rigor RR, Miller DS (2010) Rapid loss of blood-brain barrier P-glycoprotein activity through transporter internalization demonstrated using a novel in situ proteolysis protection assay. J Cereb Blood Flow Metab 30: 1593–1597. 20628400 10.1038/jcbfm.2010.117 PMC2949254

[39] Rigor RR, Hawkins BT, Miller DS (2010) Activation of PKC isoform beta(I) at the blood-brain barrier rapidly decreases P-glycoprotein activity and enhances drug delivery to the brain. J Cereb Blood Flow Metab 30: 1373–1383. 20197783 10.1038/jcbfm.2010.21 PMC2949219

